# Association of Affected Neurocircuitry With Deficit of Response Inhibition and Delayed Gratification in Attention Deficit Hyperactivity Disorder: A Narrative Review

**DOI:** 10.3389/fnhum.2018.00506

**Published:** 2018-12-18

**Authors:** Xixi Jiang, Li Liu, Haifeng Ji, Yuncheng Zhu

**Affiliations:** ^1^Shanghai Changning Mental Health Center, Affiliated Greenland Hospital of Bio-X Institute, Shanghai Jiao Tong University, Shanghai, China; ^2^Shanghai Mental Health Center, Shanghai Jiao Tong University School of Medicine, Shanghai, China

**Keywords:** attention deficit hyperactivity disorder, Tourette’s syndrome, oppositional defiant disorder, obsessive-compulsive disorder, executive function

## Abstract

The neural networks that constitute corticostriatothalamocortical circuits between prefrontal cortex and subcortical structure provide a heuristic framework for bridging gaps between neurocircuitry and executive dysfunction in attention deficit hyperactivity disorder (ADHD). “Cool” and “Hot” executive functional theory and the models of dual pathway are supposed to be applied within the neuropsychology of ADHD. The theoretical model elaborated response inhibition and delayed gratification in ADHD. We aimed to review and summarize the literature about the circuits on ADHD and ADHD-related comorbidities, as well as the effects of neurocircuitry on the executive dysfunction in ADHD.

Attention deficit hyperactivity disorder (ADHD) is a common neurodevelopmental disorder in childhood and adolescence with an overall prevalence of 6.26% in China (Wang et al., 2017) and 5.2% worldwide (Polanczyk et al., 2007). Neural impairment is associated with many profound complex issues. For example, the patients with ADHD tend to have the response delayed in many aspects such as in the development of neurological organization and neuroplasticity (Van Doren et al., 2017), network analysis and brain development (de Lacy et al., 2018), cognitive function and motor control integration (Leisman et al., 2014), synergies and motor primitives, retained primitive reflexes (Konicarova et al., 2013) and many other areas that all impinge on which is necessary to build models to help understanding the nature of neurodevelopmental disorders. As ADHD symptoms change over time, linguistic and locomotor maturation cannot reach social maturity at any developmental stage (Giertuga et al., 2017). Executive function (EF) generally refers to the cognitive ability needed to achieve goal-directed behavior. Deficits of response inhibition in neuropsychological functions exist widely in ADHD and there are four executive neuropsychological functions affected by their effective EF: self-regulation of affect-motivation-arousal (delayed gratification), working memory, internalization of speech, and reconstitution of behavioral analysis and synthesis. The executive dysfunctions in ADHD have not only been supported by Barkley (1997), but also tended to be the result in comorbid mental disorders in childhood and adolescence. Psychiatrists still encounter the difficulties in distinguishing various symptoms throughout children’s abstract thinking and emotional expressing during clinical interview. The symptoms, such as hyperactivity, impulsivity, compulsivity, irritation or motor tics, may result from similar affected regions of cerebral network. The potential overlapping affected neural network may be the main reason why ADHD is highly possible to result in comorbid mental disorders.

The heritability of ADHD was calculated to be approximately 0.76 (Coghill and Banaschewski, 2009). Today, it is known as other neurological and psychiatric diseases, that ADHD has a multifactorial etiology, in which the endogenous retroviruses of human being are also implicated resulting from a complex interaction of environmental, biological and genetic factors (Balestrieri et al., 2014). Candidate gene association studies have been focused on dopaminergic (DRD4, DRD5, DAT1, COMT); serotonergic (5-HTT, HTR2A, HTR1B); noradrenergic (ADRA2A, DBH); cholinergic (CHRNA4); central nervous system developmental pathway (BDNF, SNAP25) and other factors from the neurotransmitter receptors and signaling (Caylak, 2012). However, the most recent study on genome-wide association studies (GWAS) cannot even replicated a SNP that reached a genome-wide significance (Grimm et al., 2018). On the other hand, subsequent pathway analysis of subthreshold DNA variants from GWAS revealed that some potential genes were neurodevelopmentally involved in the expressed gene-networks. Therefore, neuroimaging endophenotypes are strategies of finding genes influencing brain structure and function closer to the action of the genes by genetic basis of EF models (Sun et al., 2018).

The imaging genetics will provide much greater power to identify the risky genes than disease status alone and it will provide a more precise understanding of ADHD’s potential of how the brain variations shaped by the genes. Many neuroscience studies have integrated structural and functional neuroimaging with genetic risk during the past decades. The advanced thoughts built the framework of imaging genetics which is to find associations of neuroimaging as endophenotypes with DNA variants as risky genes, and then use hypothesis-free whole-brain voxel-wise genome-wide to associate these studies (Wu et al., 2014). Those fore-mentioned important findings are developed from the fundamental theory of executive dysfunction and neurocircuitry in ADHD.

This review discusses the role of abnormal connections in each corticostriatothalamocortical (CSTC) circuit, which may be responsible for targeted executive dysfunction at the neuroscience level, respectively.

## Neuroscience of ADHD

The etiology of ADHD remains unclear; it is mainly considered that the disease is caused by inappropriate connection of neural network that may be associated with many different regions. The comorbidity with ADHD implies a single or multiple regional dysfunctions affecting integrated connections between regions. Because of children and adolescents’ cerebral function of differentiation and contingency involved in the neural network, especially when nervous system has been damaged, bypass can be rebuilt to compensate the damaged function called neuroplastic changes (Jehna et al., 2017). Perceptions and mutual influences of a child are more sensitive comparing to that of an adult, and a damaged perception is more likely to influence multisensory integration. Besides, children and adolescents’ neurodevelopment appears to be alternate between neural networks belonged to different systems, the myelination of primary neurons completes earlier than that of advanced central nerves. Sensory system and exercise system complete earlier than systematic integration (Krogsrud et al., 2016). Therefore, it is difficult to determine which is the main cause of the systematic dysfunction, the disease of neurological organization or mental retardation. This problem has never attracted so much attention in multidisciplinary studies. Along with a significant development on neuroscience, the principles given underlie many indirect evidences for elaboration of etiology and pathogenesis of ADHD (Roohi-Azizi et al., 2017; Karalunas et al., 2018; Van Doren et al., 2018). Neuropsychology is an interdisciplinary study between neuroscience and cognitive, and behavioral medicine is about to better explain the pathogenesis. The combination of neuropsychology and neuroimaging has been ready to come out as a highlight research on mapping endophenotypes of cognitive and behavioral characteristics.

### New Progress in Neuropsychology of ADHD

Neuropsychological studies can be less invasive and more informative on the functioning of specific neurocircuitries involved in ADHD and have more advantages than any other studies carried out by using assessment scales or quantitative tests (Pasini et al., 2007). Neuropsychology is a science to study the connection between brain function and mentality or between brain function and behavior. It is aimed to clarify different regional functions of brain and mental traits with or without impaired region of brain presented by neuropsychological test. The neuropsychological test is one of the most effective research methods. Traditional neuropsychological theory mainly projects from observation of the adults’ brain damage, neglecting the fact that the neuropsychological mechanism and characteristic of children and adolescents differ from that of adult for the aspect of brain maturation delay effect (Hoogman et al., 2017) and many ADHD patients can be self-healed by self-control enhancement with age (Karam et al., 2015). Because the neuroplasticity is flexible ahead of neurodevelopment completed, neuropsychological dysfunctions are more compensatory in childhood than in adulthood. The cortical redevelopment after damage may benefit from a highly plastic cortex in childhood, resulting in less persistent deficit than after neurodevelopmental maturity (Sharma et al., 2016). It should be emphasized that the adolescents’ neuropsychological results are quite different from adults’. The former tend would be interpreted uncertainly and limitedly. Moreover, adolescents’ psychological reactions suffer from corresponding central lesions in some cases, for example the patients with ADHD may appear to be neuropsychological reactions with relatively specific imaging study. Last but not least, another imperative characteristic is that the neuropsychology of adolescents are not independent performances, instead, it often associated with comorbid disorders (Ter-Stepanian et al., 2017). Most of the current neuropsychological researches are focusing on the EF of ADHD (Zhu et al., 2018).

#### Model of Executive Function

The neuropsychological model is set up to investigate the etiology of ADHD, its related comorbidities, and pathological mechanism as well, and to guide the clinical diagnosis, treatment and rehabilitation. Regarding the complicated connotation and advanced cognitive ability, the explanation of EF model is more efficient than any other neuropsychological models. The theory of ADHD from Barkley (1997) is widely recognized as a basic neuropsychological research of the EF of ADHD. EFs include five domains: response inhibition, working memory, cognitive flexibility, planning ability, and verbal fluidity. His model of ADHD pathogenesis postulated a link between response inhibition and working memory, which predicted the response inhibition performance on working memory but it did not predict the performance on divided attention (cognitive flexibility) and sustained attention. These data on each domains of EFs suggested the involvement of partially independent neural circuits which are involved in response inhibition, working memory and cognitive flexibility in ADHD (Pasini et al., 2007). Various impairments lead to self-control and target-seeking deficits, and we can observe these cognitive and behavioral symptoms classified in diagnostic criteria.

The above definition classifies all domains of EF of ADHD into abstract thinking, which is currently known as “Cool” EF in the cognitive and behavioral level. However, such classification unilaterally ignores the changes after the involvement of emotional response. With the development of neuropsychology, decision making encountered with emotional involvement has been defined as “Hot” EF. The “Hot” EF includes motivation and reward, emotional and cognitive process, and affective decision-making process. For example, delay gratification is characterized by emotional trait, which complements the insufficiency of the classification of “Cool” EF. Based on a large number deficits in the EF of ADHD, the impaired response inhibition and delay gratification is regarded to be core problems leading to a wide range of impairments (Pauli-Pott et al., 2014; Dalley and Robbins, 2017).

Thus, the two major domains – abstract thinking (Cool) and emotional trait (Hot) – trigger the mechanism of onset of ADHD (Zhu et al., 2018).

**FIGURE 1:**
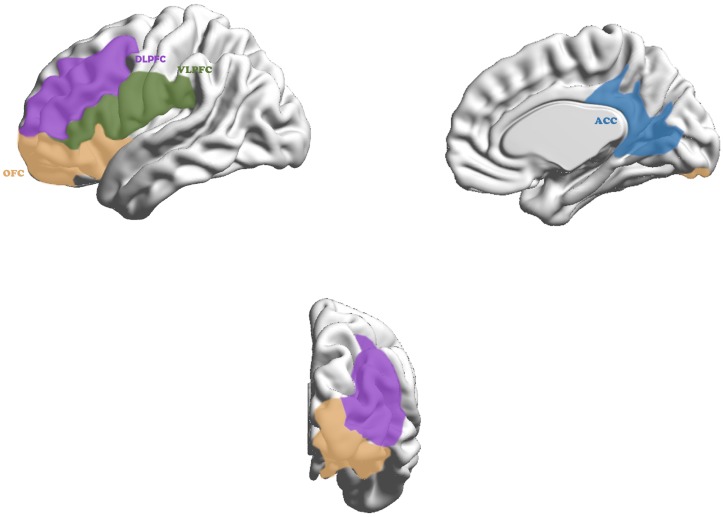
Cortex in neurocircuitry models related to executive function in ADHD. The “Cool” executive function, as a top-down process, maps from the dorsolateral prefrontal cortex (purple) and dorsal anterior cingulate cortex (blue) to the superior caudate nucleus and the “Hot” executive function, including top–down and bottom–up process, maps from ventrolateralprefrontal cortex (green) and orbitofrontal cortex (yellow) to inferior caudate nucleus.

Brain mechanism underlies these different functions shown in Figure 1: “Cool” and “Hot” EF (Antonini et al., 2015). The “Cool” EF is a top-down process from the dorsolateral prefrontal cortex (DLPFC) and dorsal anterior cingulate cortex (DACC) to the superior caudate nucleus, essentially called pure cognitive process (Bari and Robbins, 2013), which is induced by abstract or decontextualized problems. the “Hot” EF includes top-down and bottom-up processes from ventrolateralprefrontal cortex (VLPFC) and orbitofrontal cortex (OFC) to inferior caudate nucleus, the bottom-up one plays more important role than that of “Cool” EF when meeting intervention strategy for emotional stress (Krain et al., 2006).

**FIGURE 2:**
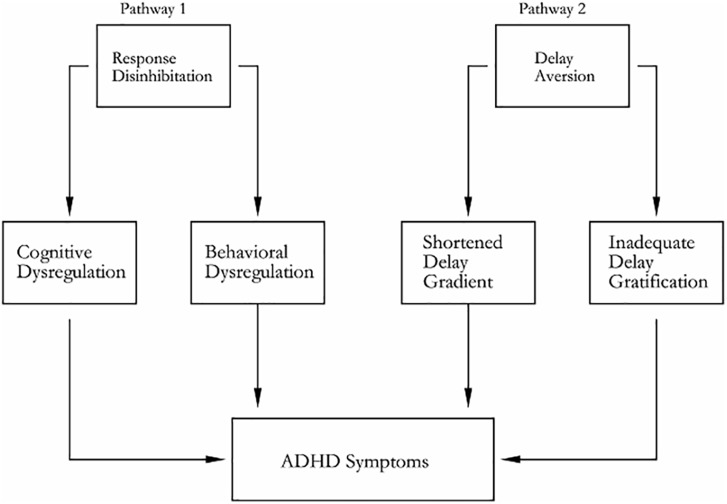
Dual pathway model of ADHD. Pathway 1 generates deficit of response inhibition for its neuropathy, which is based on the ventral and dorsal corticostriatal pathway; pathway 2 generates delay aversion for its neuropathy, which is based on the ventrolateral and orbitofrontal corticostriatal (including nucleus accumbens) pathway.

Sonuga-Barke (2002) presented dual pathway model as shown in Figure 2. Beyond the dual pathway model, evidences were obtained for the dissociation of inhibitory control and delay aversion in ADHD (Sonugabarke et al., 2010). The “Cool” and “Hot” EFs are, respectively, coincided with dual pathway model. Anomaly of fiber tracts connect between thalamus and striatum, and between thalamus and the dorsolateral prefrontal cortex DLPFC/OFC fiber tracts in ADHD (Xia et al., 2012). The deficit of response inhibition and delay aversion represented independent neuropsychological components (Yang et al., 2011). A hypoactivation of DACC and a bilateral activation of VLPFC during a counting stroop paradigm (Bush et al., 1999).

#### Response Inhibition

Special neuropsychological tasks should be designed to meet the needs of different research purposes. The response inhibition is the core element of the “Cool” EF that refers to inhibiting the reaction of irrelevant stimulus when seeking a cognitive representation of target stimulation (Szekely et al., 2017). The response inhibition is a key factor that takes effects on the process of completing a task and coordinates various psychological processes. In a narrow sense, many researchers equate the response inhibition with the EF through presenting its outcome instead of subset relationship (Kavanaugh, 2016). Because of the deficit of response inhibition, subjects usually present persistent errors, to put it into another way, subjects constantly behave what is supposed to be inhibited (Janssen et al., 2016). The persistent errors that are explained by the response inhibition theory are due to the invalid or immature inhibition mechanism. As a result, the subjects cannot suppress the tendency of strong or anticipative conflicting reaction. For example, classic Stroop test exactly tests the function of response inhibition. The test requires subjects to control the inhibitory interference against the conflict between word-color (displayed information) and identifying the meaning (supposed to be reported) according to the rules. When successfully inhibiting the conflict, the subject will complete the test. It belongs to the abstract thinking domain. ADHD patients exhibit more difficulty with response inhibition compared to typically developing youth (i.e., more commission errors, less correct responses) (Tenenbaum et al., 2018) mainly due to the dysfunction of DLPFC (sustained attention) and the DACC (divided attention) circuits. Treatment effects in ADHD are often evaluated by inhibiting prepotent responses, especially concerning functional neuroanatomical structures (Bluschke et al., 2018).

#### Delayed Gratification

In the study of the “Hot” EF, the delayed gratification is a classic paradigm of mental phenomenon. The delayed gratification refers to the tendency that subjects delay short-term satisfaction spontaneously so as to obtain long-term benefits, which demands the ability of self-regulation and self-control (Shimoni et al., 2016). For example, Delay Discounting task requires subjects to make choice between two virtual reinforcers for simulating real environment: one is high-valued but delayed reward, and the other is a low-valued but instant reward. The classic Delay Discounting task exactly presents the neuropsychological domain of delayed gratification. Neither top-down nor bottom-up process is dispensable in “Hot” EF. The development of efficient self-controlled ability is probably predicted by showing delayed gratification in the future of life. The temptation of short-term gratification will be well withstood after target acquired, provided that a necessary condition will be created for the high-value target achieved instead. More necessary conditions are provided to accomplish wonders, coordinate relationships, or adapt social maturity. ADHD patients show the tendency to prefer smaller immediate rewards over larger delayed rewards due to inefficient self-controlled ability (Norman et al., 2017). The delayed gratification is the realization of self-control and psychological maturity as well. Whether subjects could choose long-term benefits or to give up the preferring of the immediate interests depends on the emotional maturity in face of some temptation against instinctive impulse. Spontaneous neuronal activities of the patients with ADHD related to delay discounting are associated with pathophysiological characteristics of impulsive behavior (Hsu et al., 2015). Impairments in response inhibition contribute to the greater delay discounting in ADHD (Martinelli et al., 2016), therefore, not only does the delayed gratification play a key role in cognitive control, but also it becomes an important part of emotional trait in ADHD (Hoogman et al., 2017).

The observation of behavioral dysfunctions shed the light on the neuroimaging researches.

### New Progress in Neuroimaging of ADHD

Neuroimaging study, chosen to be endophenotypes between genetic inheritance and human behavior, can obtain data of disease characteristics stably and provide indirect evidence of EF association study noninvasively. According to Diagnostic and Statistical Manual of Mental Disorders (DSM-5), ADHD has been classified as a kind of neurodevelopmental disease occurs before age of 12, thus, we consider that the pathogenesis of ADHD is related with neurodevelopmental retardation. The cortex, as the upmost center of executive control system, not only needs to adjust complex cognition and emotion, but also integrates the whole neural networks rather than a single abnormal disconnection. Neuroimaging of ADHD has gone through three stages roughly over time: hypothesis of prefrontal hypofunction, hypothesis of corticostriatal pathway hypofunction and hypothesis of CSTC circuits dysfunction (Zhu et al., 2018).

#### Hypothesis of Prefrontal Hypofunction

Prefrontal cortex, as the most advanced part of cerebrum, has assembled a large number of related studies in ADHD including response inhibition and sustained attention locating within the DLPFC (Soltaninejad et al., 2015), affective symptoms locating within the ventromedial prefrontal cortex (VMPFC) (Hägele et al., 2016), divided attention locating within the DACC (Schweren et al., 2017), delay discounting and hyperactivity locating within the OFC (Yates et al., 2014) and motor control locating within the sensorimotor cortex (SMC) (Ter Huurne et al., 2017). In the previous literature published by Stahl SM, those five cerebral regions are associated with ADHD (Stahl, 2013). As a neurodevelopmental disease, the malfunction of the prefrontal cortex may explain why the above symptoms are commonly seen in ADHD.

Prefrontal dysfunction causes clinical phenotypes including hyperactivity, inattention, emotional instability and lack of planned behavior (Stahl, 2013). The hypothesis established a link between executive dysfunction and prefrontal abnormality of patients with ADHD. The early stage of MRI studies of ADHD focused on the function and volume of white matter. Castellanos et al. (2002) found that the whole brain volume of ADHD decreases by 3–5% compared to normal control group while the prefrontal lobe volume weights the most. The right prefrontal hypoactivation was reported to be found in the patients with ADHD when undergoing task for evaluating response inhibition (Monden et al., 2015). Comparatively, the prefrontal cortex cannot be activated in the patients with ADHD, and the thickness of prefrontal cortex has been implicated in the deficit of response inhibition (Stramaccia et al., 2015). Since right prefrontal cortex seems to be important in controlling response inhibition, while left dorsolateral prefrontal cortex seems crucial in modulating divided attention, these areas are deputed to be involved in the pathogenesis of neuropsychological deficits in ADHD subtypes (Pasini et al., 2007). From the theory of neural evolution, we know that the prefrontal lobe is the latest and maturest evolutionary part of brain. White matter maturation, including myelination, starts prenatally and appears to progress in an orderly manner during infancy and childhood from posterior-to-anterior, inferior-to-superior, and central-to-peripheral regions (Krogsrud et al., 2016). Human beings experience a far more complex and longer neurodevelopmental phase which provides more risks for shaping the immature brain between prefrontal cortex and subcortical structures. Changes occur at various levels – from neuroplasticity including in a given region and its connectivity to other regions, to the function of neurotransmitter systems (Andersen and Navalta, 2004).

#### Hypothesis of Corticostriatal Circuitry Abnormality

Along with further researches, the hypothesis is gradually accepted that the onset of ADHD is associated with prefrontal cortex and major striatal subregions connectivity abnormalities (Hong et al., 2015). The frontostriatal functional connectivity takes the responsibility for how information is processed by permitting transmission of signal downstream from cortex, and getting a feedback simultaneously from striatum to cortex. Many fMRI researchers have found out that these impairments can be caused by decreased neurons within prefrontal cortex and striatum of ADHD (Ortiz et al., 2015; Hauser et al., 2016; Szekely et al., 2017; Norman et al., 2018). Compared to normal controls, many statistical evidences of ADHD neuroimaging have been provided for neuropsychological deficits owing to these regions. The pathological base of ADHD correlates significantly to the abnormal circuitry. The corticostriatal circuitry abnormality plays an important role in the pathogenesis of ADHD (Hong et al., 2015).

**FIGURE 3:**
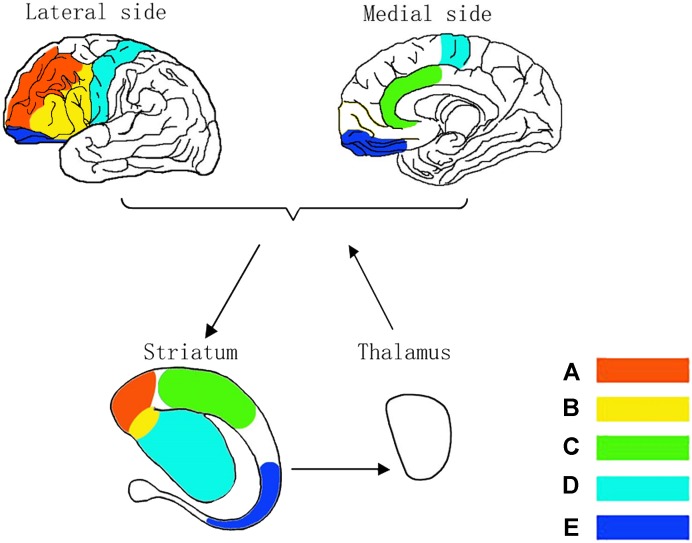
Neurocircuitry models in corticostriatothalamocortical circuits. Dorsolateral CSTC circuit for being known as sustained attention/executive function circuit **(A)**, ventrolateral CSTC circuit for emotion circuit **(B)**, anterior cingulate CSTC circuit for divided attention circuit **(C)**, motor CSTC circuit for hyperactivity circuit **(D)** and orbitofrontal CSTC circuit for impulsivity/compulsivity circuit **(E)**.

The malfunction of the prefrontal cortex corresponds with each substructure in striatum shown in Figure 3. Neural impulses originate from the DLPFC project into the superior caudate nucleus (Figure 3A), the VMPFC to the nucleus accumbens (Figure 3B), the DACC to the inferior striatum (Figure 3C), SMC to the putamen (Figure 3D) and the OFC to the inferior caudate nucleus (Figure 3E; Zhu et al., 2016). Frank et al. (2001) suggested that each of these circuits might be subdivided into various subcircuits and estimated the human prefrontal cortex to contain about 20,000 such circuits in total. Interestingly, the assumption of independent circuits implies that each corticostriatal pathway has a variety of separate channels (i.e., one for each circuit) and that each of these channels might subserve a different function (Schroll et al., 2012) as described in ADHD symptoms. Thus, it might be more fruitful to search for superordinate principles of pathway functions than for specific pathway contributions related to individual circuits (Schroll and Hamker, 2013).

#### Hypothesis of CSTC Circuits Dysfunction

Nerve signals are projected from prefrontal cortex into striatum, and then reach thalamus before return to the cortex. In detail, thalamus produces regional interaction with area-oriented cortex. The signals of CSTC circuits that pass through the striatum are able to be synapse-linked to the special part of the striatal neurons from striatum to thalamus when getting a feedback simultaneously before finally return to the initial pyramidal cells (Hauser et al., 2016). A cognitive or behavioral symptom is not just caused by a separated neural abnormality in ADHD, but also caused by the abnormalities of CSTC circuits provide a framework for further understanding of ADHD symptoms (Zhu et al., 2016).

Xia et al. (2012)found that the inattention and executive dysfunction of ADHD are highly correlated with decreased mean fractional anisotropy and volume of the tracts between prefrontal cortex, striatum and thalamus. Meanwhile, the evidence-based studies revealed the high rate of comorbidity between ADHD and Tourette’s syndrome (TS) (Groth et al., 2017), oppositional defiant disorder (ODD) (Xia et al., 2012) as well as obsessive-compulsive disorder (OCD) (Abramovitch et al., 2015). For finding out the reason, a MRI study of TS indicated that the tic severity is negatively correlated with the sensorimotor cortical thickness and caudate nucleus volume in the motor circuit (Fahim et al., 2010). On the other hand, the connectivity between the ACC and related striatum increases significantly around regional pathway, but decreases along with more distant connections (Shprecher et al., 2014). Microstructural abnormalities of thalamus and basal ganglia play an important role in the pathophysiology of TS (Li et al., 2010). ODD was found reduced in volume within the ACC and OFC (Sebastian et al., 2016), furthermore, the connection between caudate and OFC may be disrupted (Finger et al., 2011). Meanwhile, OCD symptoms may result from the increased functional connectivity between the medial thalamus and striatum with the decreased functional connectivity between the OFC and dorsomedial striatum (Jung et al., 2017). The specific strength of connectivity between the OFC and ventral caudate/nucleus accumbens decides overall symptom severity (Harrison et al., 2009). In addition, cognitive dysfunction is still linked to the DLPFC circuit abnormality in OCD (Liu et al., 2017). The hypothesis of neural disconnection of CSTC circuit underlies the disorder in both task state and resting state.

Prefrontal hypoactivity, influencing the DLPFC, ACC and related regions including striatum and thalamus, plays the role of a villain in the EF of ADHD (Dickstein et al., 2006). Each volume of CSTC circuits differs between the patients with ADHD and normal controls, so the characteristic of CSTC circuits highlights in neuropsychological research recently. The abnormal activation of CSTC circuits is the key factor leading to executive dysfunction. Dorsolateral Prefrontal Corticostriatothalamocortical (DLPFCSTC) circuit is known as the EF circuit (Stahl, 2013), while concrete neuropsychological mechanism is different. CSTC circuits connected the frontal lobe to the basal ganglia controls the response inhibition and delay aversion (Krain and Castellanos, 2006). In addition, Orbitofrontal Corticostriatothalamocortical (OFCSTC) circuit also adjusts delay discounting procedure in ADHD simultaneously (Yates et al., 2014). Neuropsychological deficits cover response inhibition and delayed aversion widely in ADHD, TS, ODD, and OCD in different ways. So researches of neuropsychology can better explain the endophenotype of ADHD (Pauli-Pott et al., 2014).

The interdiscipline of the neuroimaging and neuropsychology attracts researchers’ attention to the point that ADHD, TS, ODD, and OCD may possess the similar biological mechanism. As mentioned above, five abnormal CSTC circuits are apparently involved in ADHD, including two in ODD (the divided attention and impulsivity/compulsivity circuits; Sebastian et al., 2016) and OCD (the sustained attention/executive function and impulsivity/compulsivity circuits; Jung et al., 2017; Liu et al., 2017), and one in TS (the hyperactivity circuit; Fahim et al., 2010). The damaged range of prefrontal lobe in ADHD spreads wider than in TS, ODD, or OCD, and that quite possibly indicated the dysfunctional area of the CSTC circuits explains the high rate of disease incidence, comorbidity and multiplicity of symptoms in populations. The more circuits are affected, the more symptoms there will be. We could regard ADHD comorbidity as a special form of ADHD rather than separating it by respective diagnostic criteria.

Here are a few examples of comorbidity. Whether ADHD is with comorbid TS or not, the decrease of neural volumes and connections in the CSTC circuits are quite consistent with ADHD-related symptoms (Fahim et al., 2010). The EF deficits of ADHD-related symptoms in TS are similar due to the similar anatomical bases on the deficit of response inhibition in the “Cool” EF (Termine et al., 2016), but the same statistical significance was not found in TS without ADHD-related symptoms (Sukhodolsky et al., 2010) because of the compensating in domain of the DLPFCSTC circuit. Adolescents with ADHD-related symptoms present the deficits in the “Cool” EF that are unrelated to ODD comorbidity, however, the comorbidity is responsible for the deficits in the “Hot” EF (Antonini et al., 2015) for decreased activities in the OFCSTC circuit. Therefore, the “Cool” EF may represent the compensation mechanism in the process of neural development in motor tics, while ODD-related symptoms in component of angry and irritable affection take responsibility for the deficits in the “Hot” EF (Noordermeer et al., 2016). The activity of OFC is negatively related to the level of risk-taking (Weber et al., 2014), which has been indicated that ADHD-related symptoms such as impulsivity, curiosity and risk-taking behaviors are exactly opposite to the OCD-related symptoms characterized by evasive behaviors against risk and novel stimulus. The major academic argument is focused on the behavior of the impulsivity between OCD and ADHD. While impulsivity was classified as a primary characteristic of ADHD symptoms in DSM-5, the impulsivity of OCD had not been clarified from compulsivity (Kim et al., 2017). The interesting findings are that the decreased delay discounting tendency has been found in OCD (Sohn et al., 2014) on the opposite of delay aversion in ADHD by recognizing the neuropsychological process as a part of the “Hot” EF. Locating the mast important effect in the amygdala is another critical stage, as it associates ADHD to emotional regulation. Although those are frequently found in the patients with ADHD, DSM-5 has not yet made it into the official criteria. Our work provides neurobiological support to the inclusion of this domain in the core ADHD phenotype (Hoogman et al., 2017). Therefore, the excessive enhancement of the “Hot” EF may be a potential biomarker of OCD and ODD because of enhanced activities in the OFCSTC circuit and VLPFC circuit, respectively.

## Conclusion

The review that has been emphatically described above is focusing on the interdisciplinary interactions in ADHD, and those theories are also available in ADHD-related comorbidities. Could those studies of the EF theories help psychiatrists to better distinguish clusters of symptoms among different mental disorders? If open-minded concept is freed out of those fixed classified diagnostic criteria, many interesting phenomena are supposed to be found according to the independent symptoms.

The neurocircuitries that constitute the CSTC circuit provide a framework for bridging gaps between neuroimaging and EF in ADHD (Zhu et al., 2016), but it has been difficult to identify the mechanisms for regulating abstract thinking and emotional responding from the understanding of ADHD comorbidity with TS, ODD, and OCD. Research based on “Cool” and “Hot” executive functional theory and circuit models, which are considered as applied response inhibition and delay aversion, respectively, within the neuropsychological view of ADHD, has shed the light on emotional responding before and after decontextualized stimuli (Zhu et al., 2018).

## Perspectives

It is believed that more and more evidence-based medicines would be found in ADHD and its related comorbidities with the deepening of the scientific researches and interdiscipline between neuroimaging and neuropsychology after being enlightened by such theories. Further researches are recommended to be carried out on the heritability, stability, specificity and familial genetic overlap of the aforementioned neuropsychological traits. The verification and identification of neuropsychological traits may offer a previous contribution to molecular genetic studies, pharmacogenomics, and clinical management of ADHD patients.

## Limitations

However, systematic errors can’t be avoided in this kind of review even with careful searching strategy. We did not evaluate the quality of these cited references while being presented their results as many qualitative descriptions. These reviewed papers, to some extent, were demonstrated depending on the authors’ viewpoint of ADHD. Furthermore, we noted that vast majority of neuropsychological data were measured by comparing to statistical significance between disease groups and typical development groups in the purpose of their researches, and then a relevant conclusion was drawn in those ways but neglecting the comparison between diseases. Those limitations might make the conclusions ambiguous.

## Author Contributions

XJ wrote the paper. LL and HJ contributed to the overall design of the article structure. YZ wrote the protocol, managed study supervision and critical revision of the manuscript for important intellectual content.

## Conflict of Interest Statement

The authors declare that the research was conducted in the absence of any commercial or financial relationships that could be construed as a potential conflict of interest.
